# *In-Vitro* Helix Opening of *M. tuberculosis oriC* by DnaA Occurs at Precise Location and Is Inhibited by IciA Like Protein

**DOI:** 10.1371/journal.pone.0004139

**Published:** 2009-01-07

**Authors:** Sandeep Kumar, Aisha Farhana, Seyed E. Hasnain

**Affiliations:** 1 Laboratory of Molecular and Cellular Biology, CDFD, Hyderabad, India; 2 Institute of Life Sciences, University of Hyderabad, Hyderabad, India; 3 Department of Biochemistry, University of Hyderabad, Hyderabad, India; 4 Jawaharlal Nehru Centre for Advanced Scientific Research, Jakkur, Bangalore, India; University of California Merced, United States of America

## Abstract

**Background:**

*Mycobacterium tuberculosis* (*M.tb*), the pathogen that causes tuberculosis, is capable of staying asymptomatically in a latent form, persisting for years in very low replicating state, before getting reactivated to cause active infection. It is therefore important to study *M.tb* chromosome replication, specifically its initiation and regulation. While the region between *dnaA* and *dnaN* gene is capable of autonomous replication, little is known about the interaction between DnaA initiator protein, *oriC* origin of replication sequences and their negative effectors of replication.

**Methodology/Principal Findings:**

By KMnO_4_ mapping assays the sequences involved in open complex formation within *oriC*, mediated by *M.tb* DnaA protein, were mapped to position −500 to −518 with respect to the *dnaN* gene. Contrary to *E. coli*, the *M.tb* DnaA in the presence of non-hydrolysable analogue of ATP (ATPγS) was unable to participate in helix opening thereby pointing to the importance of ATP hydrolysis. Interestingly, ATPase activity in the presence of supercoiled template was higher than that observed for DnaA box alone. *M.tb* rRv1985c, a homologue of *E.coli* IciA (Inhibitor of chromosomal initiation) protein, could inhibit DnaA-mediated *in-vitro* helix opening by specifically binding to A+T rich region of *oriC*, provided the open complex formation had not initiated. rIciA could also inhibit *in-vitro* replication of plasmid carrying the *M.tb* origin of replication.

**Conclusions/Significance:**

These results have a bearing on the functional role of the important regulator of *M.tb* chromosomal replication belonging to the LysR family of bacterial regulatory proteins in the context of latency.

## Introduction

Replication in eubacteria is initiated when DnaA, an initiator protein, binds to DnaA boxes located within the origin of replication (*oriC*) sequence [1]. Initiation of replication in *E. coli* proceeds with the binding of DnaA protein to *oriC* [2] and leads to opening of 13-mer region, which is followed by entry of DnaB helicase to form the prepriming complex [3]. In many bacteria either or both the 3′ and 5′ flanking regions of the *dnaA* gene exhibit *oriC* activity, thereby conferring the ability to replicate autonomously. In *Bacillus subtilis*, both the 5′ and 3′ flanking regions of *dnaA* act as *oriC* [4], whereas in *Mycobacterium tuberculosis* (*M.tb*), *M. bovis* [5] and *M. smegmatis* [6], [7], [8], only the 3′ flanking region provides *oriC* function. There are five DnaA-binding sites in the *oriC* region of *E. coli*, referred to as R boxes, to which both active ATP-DnaA and inactive ADP-DnaA proteins bind with equal affinity [9], [10]. There are additional initiator binding sites in the *oriC*, region referred to as I sites, to which only DnaA-ATP can bind [11].

DnaA protein binds with nearly equal affinity to ATP and ADP. In *E. coli* the function of ATP appears to be allosteric and the non-hydrolysable analogue ATPγS can replace ATP in helix unwinding [12]. For opening of the DNA duplex multiple DnaA proteins, complexed with ATP, bind to *oriC* and melt the DNA unwinding element (DUE). ADP bound form of DnaA is inactive for replication initiation, forming an important level of regulation at the origin.

The *E. coli* IciA protein (Inhibitor of Chromosome Initiation) blocks initiation at very early stage *in-vitro* by binding specifically to A+T rich region of *oriC* [12], [13]. Binding of IciA blocks the opening of A+T rich region mediated by DnaA and HU (Histone like protein) or integration host factor (IHF) protein and this inhibition of strand opening by IciA does not affect binding of DnaA and IHF (or HU) protein to their respective binding sites [14]. IciA contains helix turn helix motif at the N terminal region and shows homology to LysR family of prokaryotic transcription regulators [12]. IciA has also been implicated in binding to A+T rich regions within the plasmid *ori* sequence and the copy number of the F plasmid is increased in *iciA* deletion mutant [15]. IciA also shows higher binding preference for curved DNA [16]. Further, IciA is involved in regulation of *nrd* gene encoding ribonucleoside diphosphate reductase [17], activating *dnaA* gene [18] and has recently been shown to also regulate the *yggA* gene encoding the arginine exporter [19].

**Figure 1:**
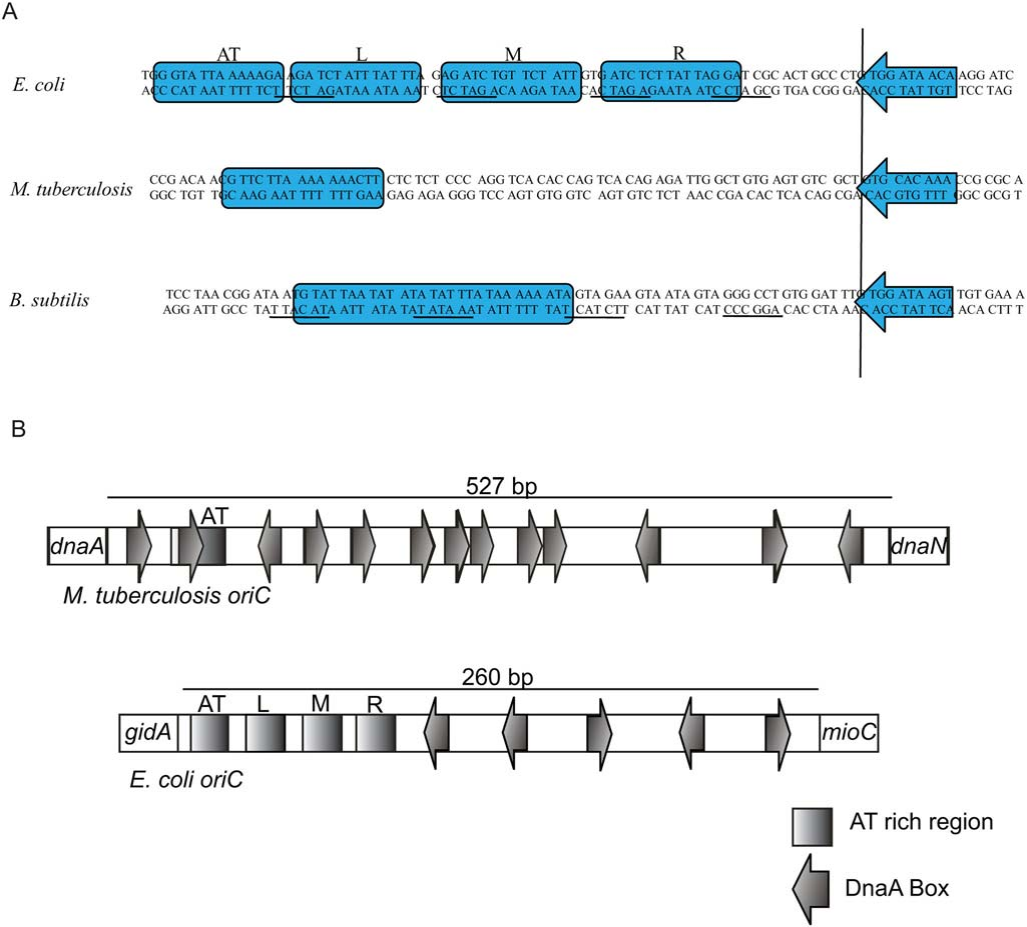
A) Alignment of A+T rich regions from *E. coli*, *M. tuberculosis* and *B. subtilis*. These regions were aligned using adjacent DnaA box (shaded arrow) to A+T rich regions. Shaded boxes represent A+T rich cluster of *E. coli*, *M. tuberculosis* and *B. subtilis* respectively. Underlined regions in *E. coli* and *B. subtilis* represent potential DnaA-ATP boxes. L, M and R represent left, right and middle 13-mers. B) Illustration showing the organization of *oriC* region of *M.tb* and *E. coli*. AT represent AT rich region (rectangle) and the arrows represent DnaA boxes. The direction of arrows represents the orientation of these boxes.

*M.tb* maintains itself in two physiologically distinct growth states – an active replicative state and a non-replicative persistent state [20]. In persistent state, the bacterium is metabolically active, but shows no multiplication for extended periods, only to revive later and multiply to cause infection [21]. The genetic elements responsible for the replication process in *M.tb*, specifically its initiation and regulation, are not known. In *M.tb*, the DNA fragments bearing the *dnaA-dnaN* intergenic region function as *oriC* [5]. Upon comparison of the *oriC* region of *E. coli*, *M.tb* and *B. subtilis* (Figure 1A) it appears that *E. coli* has three A+T rich 13 mers [1], *B. subtilis* has a 27 mer [4] which is exclusively rich in A+T residues, but *M.tb* has only one A+T rich 15 mer region [5], [22]. It should also be noted that *E. coli* has only 5 DnaA boxes (Figure 1B) whereas *M.tb* has 13 such boxes. In addition, both *E. coli* and *B. subtilis* have DnaA-ATP boxes (Figure 1A), however in *M.tb* such boxes are not present [23]. One more unusual observation reported for *M.tb* is the requirement of hydrolysis of ATP for rapid oligomerization of DnaA on *oriC* [23]. It should also be noted that *E. coli* possesses only five DnaA boxes, whereas *M.tb* has 13 presumptive DnaA box sequences that bear little sequence similarity to any of the *E. coli* DnaA boxes [5], [8]. DnaA protein of mycobacteria has been shown to bind to at least some of these boxes [24], [25]. These studies suggest that the replication origin site in *M.tb* is very complex thereby making it interesting to study the mechanism of DNA replication and its regulation in *M.tb*.

Given the clinical significance of persistence within the macrophages, it is important to identify and characterize the events involved in *M.tb* replication initiation and the negative effectors of replication initiation. We describe the interaction between *M.tb* DnaA protein and the *M.tb oriC*, including mapping the nucleotide sequences involved in DNA opening, and the requirement of ATP hydrolysis in this process. We additionally show the ability of *M.tb* IciA like protein, coded by *Rv1985c*, to block DnaA mediated helix opening and the eventual DNA replication by specifically interacting with A+T rich sequences present within the *oriC* region.

## Results

### DnaA protein shows higher ATPase activity in the presence of supercoiled template

**Figure 2:**
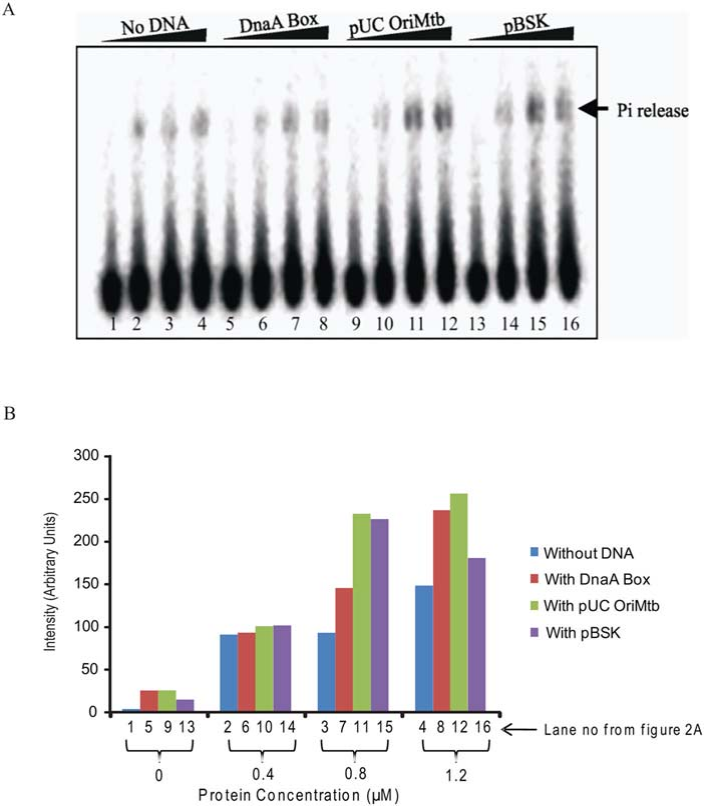
A) DnaA protein (2 μM) was incubated with 16 nM [γ^32^P]ATP for 30 min at 0°C in buffer C. Lanes 1–4: 0, 0.4, 0.8 and 1.2 μM respectively, of DnaA protein without any DNA; lanes 5–8: 0, 0.4, 0.8 and 1.2 μM respectively of DnaA protein with DnaA box; lanes 9–12: 0, 0.4, 0.8 and 1.2 μM respectively of DnaA protein with 550 ng of pUC_OriMtb and lanes 13–16: 0, 0.4, 0.8 and 1.2 μM respectively of DnaA protein with 550 ng of pBSK II. B) The phosphate released in ATPase assay was quantified using Typhoon Variable Mode Imager and Image Quant Software (Amersham).

In order to determine the preference, if any, of *M.tb* DnaA for a given form of DNA template, DnaA protein activity was measured in terms of ATPase activity. Recombinant DnaA protein expressed in *E. coli* was refolded after its purification under denaturing conditions and assayed for ATPase activity. ATPase activity was assayed either in the absence of DNA, or in the presence of linear DNA, or supercoiled pUC_OriMtb, or non-specific supercoiled template pBSK II. As could be seen from the densitometric scanning of the gel, ATPase activity in the presence of DnaA box (Figure 2, lanes 5–8) is expectedly higher than in the absence of DNA (Figure 2, lanes 1–4). However, ATPase activity increases significantly in the presence of supercoiled pUC_OriMtb (lanes 9–12) and pBSK II (lanes 13–16) (Table 1). The ATPase activity is a direct function of the concentration of rDnaA protein with maximal activity at 0.8 μM after which it stabilizes. These results while confirming that the refolded rDnaA protein is enzymatically active, also confirm that DnaA has very weak intrinsic ATPase activity which however increases in the presence of supercoiled DNA independent of whether *M.tb oriC* is present or not.

**Table 1:** Bacterial strains, plasmids and oligonucleotide primers used in the current study. Nucleotides in bold represent the restriction enzyme sequence appended to the primers to enable directional cloning in pET28a/pUC18 vector.

Bacterial Strains	Relevant characteristics	Source/ref.
**Strains**		
*E. coli* DH5α	*supE44* Δ*lacU169* (Φ80lacZΔM15) *hsdR17 recA1 endA1* and *endA1 gyrA96 thi-1recA1β*	Invitrogen
*E. coli* BL21(DE3)	F^−^ *omp*T *hsd*S_B_(r_B_^−^m_B_^−^) *gal dcm* (DE3)	Invitrogen
*E. coli* BL21(DE3) PlysS	F^−^ *omp*T *hsd*S_B_(r_B_^−^m_B_^−^) *gal dcm* (DE3) pLysE (Cam^R^)	Invitrogen
*M. bovis* BCG pasteur	Vaccine Strain, Pasteur Institute	AstraZeneca, India
**Plasmids**		
pUC18	Ampicillin resistant; multicopy plasmid with a ColE1 – type replicon	Fermentas
pET28a	Expression Vector (kanamycin resistant)	Novagen
pETDnaA	pET28A derivative carrying *M.tb DnaA (Rv0001)*	This work
pETIciA	pET28A derivative carrying *M.tb IciA (Rv1985c)*	This work
pUC_OriMtb	pUC18 derived carrying intergenic region between *dnaA* and *dnaN*	This work
pBSK II	Ampicillin resistant multicopy plasmid with a ColE1 type origin sequence	Strategene
**Oligonucleotide primers**		
IciAF	GGAATT**CATATG**GTGGATCCGCAGCTTGA	
IciAR	GC**AAGCTT**TCAACCCGGTCGGCGGCGGC	
DnaAF	GGAATT**CATATG**ACCGATGACCCCGGTTC	
DnaAR	GC**AAGCTT**CTAGCGCTTGGAGCGCTGAC	
MtbOriF	GC**AAGCTT**CGCTAGCACGGCGTGTTCTT	
MtbOriR	GC**GGATCC**CCACGAAACGTCAAGTCGGTGA	
DnaA box 8-9Fw	ACCAGACTGTCCCCAAACTGCACACCCTCT	
DnaA box 8-9Rv	AGAGGGTGTGCAGTTTGGGGACAGTCTGGT	
Ori F1	TTCTTCCGACAACGTTCTTAAAAAAACTTCTCTA	
Ori R1	TAGAGAAGTTTTTTTAAGAACGTTGTCGGAAGAA	
SeqOriR1	TCTTGGTGCAGGTCGACGTCGGTCGGAGT	
SeqOriR2	ACCGCCGGGACTGTATGA	
SeqOriR3	GTTTTCCCAGTCACGAC	

### Open complex is formed near the A+T rich repeat

*oriC* region of *M.tb* is very complex and is different from its *E. coli* counterpart (Figure 1). The *M.tb oriC* has 13 imperfect DnaA boxes, which bear little sequence homology to *E. coli* DnaA boxes and also lack distinct A+T rich nucleotide repeat which is however present both in *E. coli* and *B. subtilis* at the 3′ end of *dnaA* gene, and is thought to be the site for helix opening. Given this complexity of *M.tb oriC*, DNA sequences involved in open complex formation were therefore mapped by primer extension analyses by KMnO_4_ probing. Permanganate is a very strong oxidant and thus reacts with the base moiety of DNA. Unlike DNase I, KMnO_4_ generally does not modify naked double stranded DNA. However KMnO_4_ selectively oxidizes unpaired pyrimidines, especially thymine residues, in single stranded DNA and in helically distorted duplex DNA. The most reactive site of the attack is 5, 6 double bond of the thymine ring. This attack can occur either from above or below the plane of the ring. But in native B form DNA this kind of attack is strongly hindered. The susceptible bond lies within the stacked array of bases under the DNA backbone within the major groove of DNA. Thus out of plane attack is just not possible as it is hindered by both the backbone and the adjacent bases. This accounts for the high selectivity of KMnO_4_ for single stranded DNA. The initial stable product of the attack on thymine is glycol (diol form). Oxidized pyrimidines prevent primer extension by the DNA polymerase beyond the modified residues. This technique is routinely used for the study of replication complexes.

**Figure 3:**
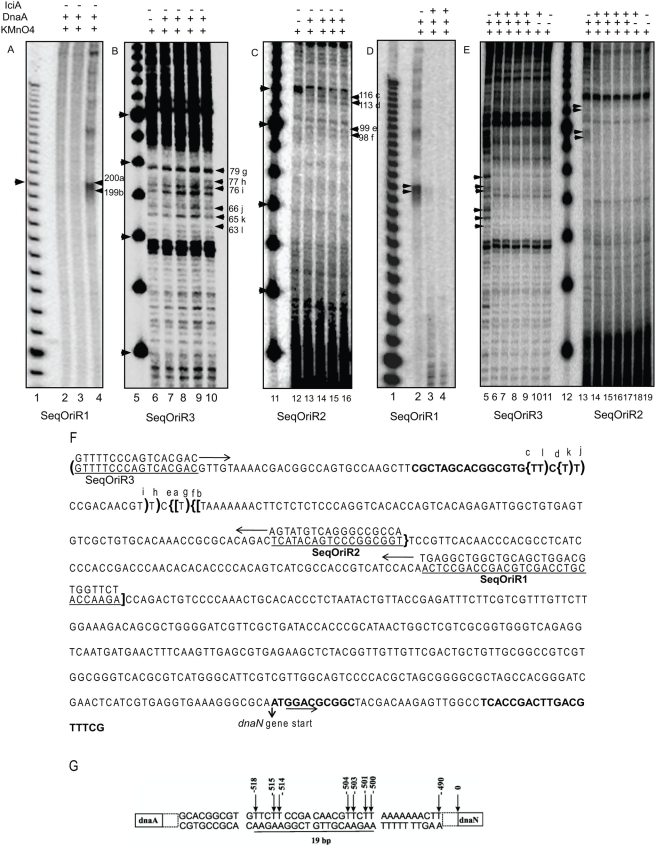
*M.tb* helix opening by rDnaA occurs near position −500 to −518 within the A+T rich region and this is inhibited by rIciA. pUC_OriMtb was used as a template for helix opening in the presence of increasing amounts of rDnaA with γ^32^P labeled SeqOriR1 and the primer extension products were fractionated on 6% sequencing gel. (A) SeqOriR1 primer reads pUC_OriMtb from bottom. Lanes 2, 3 and 4 show KMnO_4_ probing in the presence of 25 ng, 50 ng and 75 ng DnaA. These and other primer extension products of various sizes were designated as 200 nt (a), 199 nt (b) and so on and are summarized in Figure 3F. (B) The upstream primer SeqOriR3 reads pUC_OriMtb from the top and anneals at position −40 of pUC18 vector backbone. The different lanes are: lane 6, no DnaA protein; lanes 7–10: 0.075, 0.1, 0.2 and 0.3 μg of rDnaA. Extension products of 79(g), 77(h), 76(i), 66(j), 65(k) and 63(l) nucleotides could be seen. (C) Primer SeqOriR2 (downstream primer) also reads pUC_OriMtb from the 3′- end. After KMnO_4_ modification and PCR amplification with γ^32^P labeled SeqOriR2, the primer extension products were fractionated on a 15% sequencing gel. Lane 12, control DNA where no DnaA protein is added; lanes 13–16: 0.075, 0.1, 0.2 and 0.3 μg of rDnaA. Extension products of 116(c), 113(d), 99(e) and 98(f) nucleotides could be seen. Non-specific extension products (ns) were also seen in all lanes even in control DnaA free lane.10 bp ladder was used as DNA molecular size marker (lane 1, 5 and 11) and shown on the left. (D) The reaction was carried out using 0.2 μg of DnaA protein. Helix opening was monitored by primer extension using SeqOriR1 on a 6% sequencing gel. The different lanes are: lane 2, without rIciA; lane 3–4: increasing amounts (0.2 μg and 0.4 μg) of IciA protein. Arrows correspond to the extension products of 200 and 199 nucleotides. (E) Primer SeqOriR3 (lanes 5–11) and SeqOriR2 (lanes 13–19) were used to monitor helix inhibition mediated by rIciA. All the lanes from 5–9 and 13–17 have 0.2 μg of DnaA protein; Lanes 6–9 and 14–17 have increasing amounts (0.2, 0.3, 0.4 and 0.5 μg) of IciA protein; lanes 10 and 18 have 0.5 μg of IciA protein; lanes 11 and 19 have no DnaA or IciA protein; and lane 12 represents 10 bp marker. Arrows on the left correspond to extension products of 79, 77, 76, 66, 65 and 63 nucleotides with primer SeqOriR3 and 113, 99 and 98 nucleotides with primer SeqOriR2. (F) The nucleotide sequence of the entire *oriC* region of *M.tb*. Letters underlined represents various primers. Amplification products obtained by primer SeqOriR1 are marked by [ ] brackets. “]” bracket represents start of primer extension product and “[” bracket represent end of the primer extension product. It could be noted that primer extension stops at T residue which is modified by KMnO_4_. The small letters “a” and “b” represent 200 nt and 199 nt band. Similarly the amplification products obtained by primer SeqOriR2 (direction of primer extension product is shown by arrow) are marked by {} bracket and the modified T residues “{” mapped by this primer are indicated by c, d, e and f which represent 116, 113, 99 and 98 nucleotide bands. Likewise the amplification products obtained by primer SeqOriR3 are marked by () bracket. Here “(” bracket marks the start of extension and “)” bracket marks the end of extension product. The modified T residue is shown by g, h, i, j, k and l which represent 79, 77, 76, 66, 65 and 63 bp bands respectively. Also the start of dnaN gene is indicated by an arrow. (G) KMnO_4_ reactive pyrimidines within the A+T rich *oriC* of *M.tb*. About 19 bp stretch of pUC_OriMtb becomes sensitive to KMnO_4_ modification (the reactive pyrimidines are indicated by arrow).

For our helix-opening assay increasing amounts of DnaA protein (0.025–0.3 μg) were incubated in presence of 5 mM ATP with supercoiled pUC_OriMtb, as described. Primer SeqOriR1 annealed between position −292 to −320 of template strand (Figure 3A), primer SeqOriR2 annealed between positions −402 to −420 of the template strand (Figure 3C) and primer SeqOriR3 annealed at position of −40 of pUC18 (Figure 3B). Primer extension reaction carried out using SeqOriR1 and SeqOriR2 would therefore enable read outs from bottom (downstream) while SeqOriR3 will give readouts from top (upstream). The extension products were then fractionated on a standard (6% or 15% as shown in the legend) urea sequencing gel (Figure 3A, B and C). Helix opening could clearly be detected in the presence of 0.075 μg (Figure 3A, lane 4) of rDnaA protein but barely when 0.025 μg or 0.050 μg (Figure 3A, lanes 2–3) of rDnaA was used and this was evident from the presence of extension products (lane 4) of 199 nucleotides(a) and 200 nucleotides(b) corresponding to position −500 and −501 from the start of the *dnaN* gene. To further pinpoint the extent of helix opening another primer SeqOriR2 was utilized and the extension products were fractionated on 15% urea gel. As can be seen (Figure 3C, lanes 12–16) extension products corresponding to 98, 99, 113 and 116 nucleotides designated as f, e, d and c respectively, could be observed which correspond to position −500, −501, −515, −518 from the start of *dnaN* gene. Primer SeqOriR3 annealed at position of −40 of pUC18 and generates extension products (Figure 3B, lanes 6–10) of 63(l), 65(k), 66(j), 76(i), 77(h) and 79(g) nucleotides which represent position −518, −515, −514, −504, −503 and −501 respectively, from start of *dnaN* gene. Irrespective of the primers used, the extension products appeared as a function of concentration of DnaA protein with 0.2 μg (200 nM) being the most efficient after which there was no further concentration effect. These mapping data, generated with different primers, are summarized in Figure 3F. To conclude, our results reveal that a 19 bp stretch of *M.tb oriC* becomes sensitive to KMnO_4_ (Figure 3G) thereby demonstrating, for the first time, that in *M.tb* the duplex opening occurs near position −500 to −518 (from start of *dnaN* gene) which lies within the A+T rich region.

### IciA inhibits helix opening

IciA, in addition to other functions, is a known inhibitor of *E. coli* chromosome replication initiation *in-vitro*. *M.tb* ORF *Rv1985c* displays 35.8% sequence identity to *iciA* of *E. coli*. Analysis of secondary structure (data not shown) also demonstrated that both IciA of *E. coli* and the putative *M.tb* IciA (Rv1985c) could be possibly functionally similar. Therefore, we analyzed the inhibitory effect of *Mtb iciA*, if any, on open complex formation. Helix opening reaction was carried out in the presence of increasing concentrations of recombinant purified IciA protein. 200 nM of rDnaA protein was used as this amount was earlier observed to be sufficient for helix opening. The appearance of primer extension products of 199 and 200 nucleotides long when primer SeqOriR1 was used (Figure 3 D, lane 2) or four extension products of 98, 99, 113 and 116 nucleotides, when the reaction was carried out using downstream primer SeqOriR2 (Figure 3E, lane 13), or six extension products of 63, 65, 66, 76, 77 and 79 nucleotides when the reaction was carried out using upstream primer SeqOriR3 (Figure 3E, lane 5), is a reflection of helix opening. Once the same reaction was carried out in the presence of purified rIciA protein these extension products could not be seen (Figure 3D and E, compare lanes 3, 4 with lane 2 and lanes 6, 7, 8, 9 with lane 5 and lanes 14, 15, 16, 17 with lane 13). Furthermore, the inhibitory effect of rIciA was a direct function of its concentration. Interestingly, the inhibition by IciA was observed only when it was added before the addition of DnaA protein, but when IciA was included 10 min after incubation at 37°C, to allow open complex formation, it failed to inhibit helix opening (Figure S1). These results suggest that once the helix opening has been initiated by the binding of DnaA protein to *oriC* and the 13- mer region has opened, IciA protein cannot block formation of the open complex thereby demonstrating that IciA protein can block open complex formation by possibly binding directly to the *oriC* sequences.

### ATPase activity is essential for open complex formation

**Figure 4:**
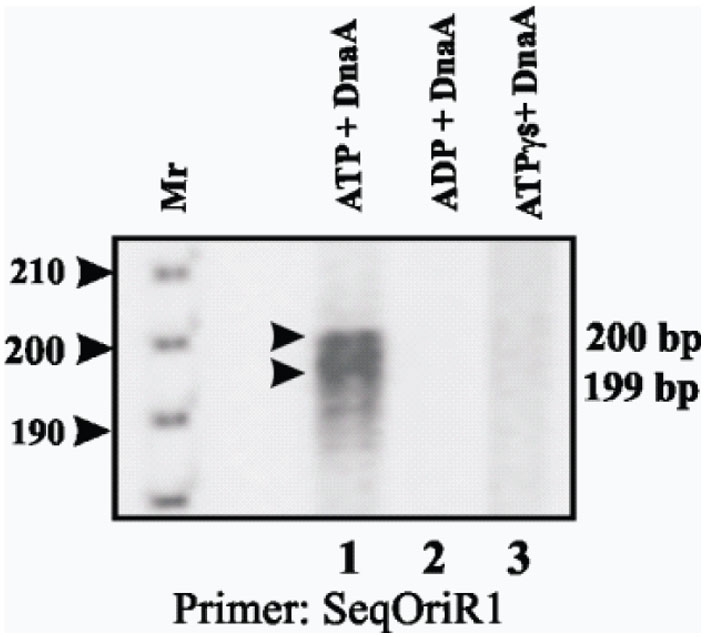
ATP hydrolysis is essential for helix opening by rDnaA. Helix opening assay was carried out as described earlier with SeqOriR1 as primer and the primer extension products were resolved on 6% sequencing gel. The reaction was carried out using 0.75 μg of rDnaA and 5 mM ATP (lane 1), 5 mM ADP (lane 2) and 5 mM ATP γ S (lane 3). 100 bp DNA ladder was used as a DNA molecular size marker (lane Mr). The markers on the right represent the size of the extension products.

Having mapped the nucleotides (within the *oriC* region of *M.tb*) involved in opening of the duplex DNA, we investigated the requirement of ATP hydrolysis and also whether other hydrolysable and poorly hydrolysable analogues of ATP could provide the necessary energy to drive this process. The *E. coli* DnaA protein has a very weak ATPase activity but the intrinsic ATPase activity of *M.tb* DnaA promotes rapid oligomerization of DnaA on *oriC* and both ATP binding and ATP hydrolysis are required for rapid oligomerization of DnaA on *oriC* [23]. We therefore carried out helix opening reaction with 5 mM of ATP, ADP and ATPγS (Lithium salt). After oxidation with 8 mM KMnO_4_ the primer extension products were fractionated as usual using 6% urea gel. Only when 5 mM ATP (Figure 4, lane 1), but not when ADP (lane 2) or ATPγS (lane 3) was used as energy donor could rDnaA bring about helix opening as could be seen from the appearance of the expected 200/199 nucleotides primer extension product. These results while highlighting the difference between *M.tb* and other bacteria, directly support the role of ATP in helix opening, which is a prerequisite for replication initiation.

### IciA inhibits DNA replication

**Figure 5:**
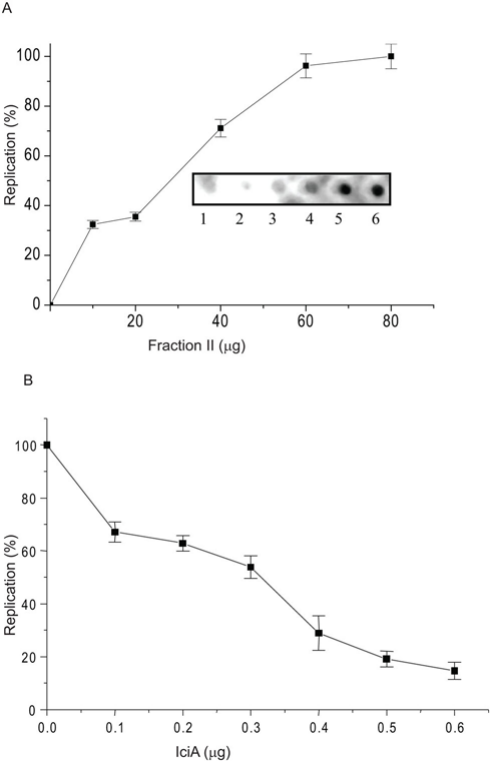
DNA replication from pUC_OriMtb is supported by *M. bovis BCG* crude extracts (Fraction II) and is inhibited by rIciA. (A) DNA replication was carried out with ammonium sulphate fractionated crude cell lysate (Fraction II), as described in Materials and Methods, in the presence of increasing concentrations (0 to 80 μg) of *M. bovis BCG* fraction II. Lane 1, 2, 3, 4, 5, 6 and 7 represent 0, 10, 20, 40, 60 and 80 μg of fraction II. The replication products were TCA precipitated and blotted (Biorad) on a nylon membrane and after densitometric scanning (inset) the values were plotted for different fractions. (B) DNA replication mediated by *M. bovis BCG* extract (80 μg) was assayed in the presence of increasing concentrations (0 to 0.6 μg) of purified rIciA.

Having shown the ability of rIciA to inhibit helix opening *in-vitro*, experiments were designed to assess the ability of rIciA to actually inhibit DNA replication by using a reconstituted replication system. *M. bovis BCG* fraction II which supports *in-vitro* replication of DNA from *M.tb oriC* (manuscript under preparation) was utilized. Quantitation of the radioactivity incorporated as a function of DNA replication reveals that maximal DNA synthesis occurs in the presence of 80 μg of fraction II (Figure 5A). Therefore this concentration of fraction II was selected to test whether rIciA could inhibit DNA replication *in-vitro* in the presence of increasing amounts of rIciA. DNA replication assay was therefore repeated except that rIciA protein was added to the assay mix before the addition of *M. bovis BCG* replication competent fraction II. As could be seen in Figure 5B, DNA replication is inhibited as a direct function of its concentration. In the presence of 0.6 μg of rIciA protein only 10% replication activity could be seen. These results directly point to the ability of rIciA to act as an inhibitor of DNA replication.

### IciA binds to A+T rich region of M.tb oriC

**Figure 6:**
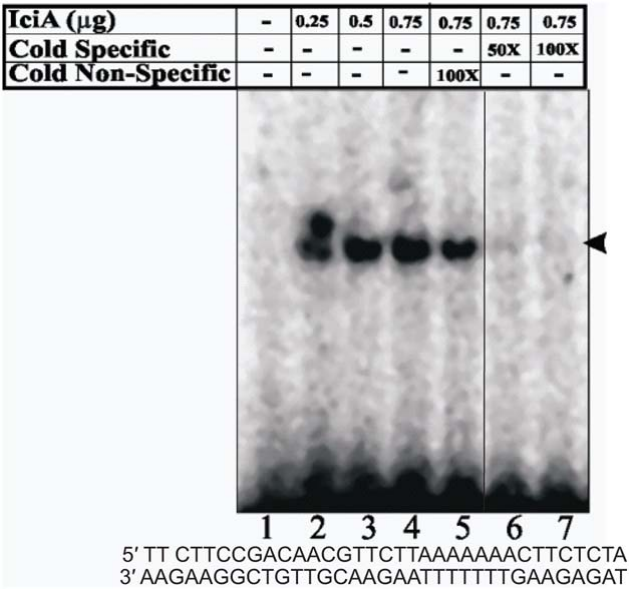
rIciA binds specifically to the A+T oligonucleotide derived from the *oriC* region of *M. tuberculosis*. Increasing amounts of IciA protein was used in electrophoretic mobility shift assays. The different lanes are: lane 1, 0 ng; lane 2, 250 ng; lane 3, 500 ng; lane 4, 750 ng ; lane 5, 100× cold non specific competitor; lane 6, 50× and lane 7 100× of cold homologous competitors. Specific DNA protein complex is indicated by an arrow. The sequence of the oligonucleotide used is given below the gel.

The results presented so far clearly suggest that rIciA is able to block helix opening (Figure 3D and E) and consequent DNA replication (Figure 5) only when it encounters the *oriC* sequence before DnaA protein has initiated helix opening thereby pointing to a possible *ori* specific DNA binding activity of rIciA protein. *M.tb oriC* is located within a small patch of A+T rich sequence which was earlier mapped as the site for helix opening (Figure 3A, B and C). Having identified the nucleotides (Figure 3F) involved in *in-vitro* helix opening, oligonucleotides corresponding to this region were used to determine DNA-protein interaction involving IciA. Electrophoretic mobility shift assays were carried out using this A+T rich *oriC* element, rIciA and huge excess (1 μg) of poly (dI/dC). Results clearly show that IciA protein binds to A+T rich region (Figure 6, lanes 2–4). That this binding is specific is clearly evident from homologous and heterologous cold competition assays. Even in 100-fold molar excess of non-specific competitor DNA, the DNA-protein complex is not abrogated (Figure 6, lane 5); whereas the DNA-protein complex completely disappears in the presence of 50 fold (lane 6) and 100 fold (lane 7) molar excess of specific homologous cold competitor DNA. These results demonstrate that IciA specifically binds to A+T rich region of the *oriC* and the inhibitory effect of IciA on the DNA helix opening (Figure 3D and E) and DNA replication (Figure 5) is a likely consequence of this *oriC*:IciA interaction.

## Discussion

Regulation of DNA replication is a very critical process mediating a switch between active and latent phase of *M.tb*. In the present study we focused on two critical proteins, the initiator (DnaA) and a putative inhibitor of replication (IciA), involved in DNA replication. The initiator protein, DnaA, is central for bacterial replication from chromosomal origin, *oriC*. In *E. coli*, initiation of replication starts when DnaA specifically recognizes nine base pair consensus sequence, termed DnaA box within the *oriC* region. *E. coli* has five such DnaA boxes in the *oriC* region, but *M.tb oriC* region has 13 such DnaA boxes. Also the *oriC* of *M.tb* lacks a distinct A+T rich repeats and the binding of DnaA to all 13 DnaA boxes is not simultaneous. It has been proposed that DnaA first binds to a few high affinity DnaA boxes followed by binding to low affinity DnaA boxes to form a productive DnaA oriC initiation complex [23]. This oligomerization results in a local unwinding of the DNA double helix at −500 and −518 relative to start of *dnaN* gene.

Earlier studies used P1 nuclease for mapping helix opening of a supercoiled plasmid [1], [14] or KMnO_4_ probing for distorted B form of DNA [26], [27]. We have used potassium permanganate (KMnO_4_) probing assay to monitor *in-vitro* opening of the DNA helix. Using KMnO_4_ probing assay we were able to determine the locus/site of opening of the double helix in *M.tb oriC*. Our helix unwinding assays reveal that DnaA mediated helix melting occurs just adjacent to a stretch of A residues within the 19 bp core of the *oriC*.

*E. coli oriC* also carries I sites, which are specific for DnaA bound to ATP. *M.tb oriC* however lacks such sites [23] and the orthologues/analogues of *E. coli Hda*, which stimulate intrinsic ATPase activity of the DnaA are also absent [28]. IHF (integration host factor) and Fis proteins which are involved in DNA bending are absent in *M.tb* [28]. *E. coli* has two histone like genes; *huα* and *huβ*, whereas *M.tb* and *M. leprae* have only one *hu* gene denoted as *hupB*. The *M. leprae* HU protein has been shown to be associated with adhesion to Schwann cells. These arguably point to the differences in the regulation of replication in *M.tb* from *E. coli*. Our results indeed show that only the ATP bound form of DnaA is active for helix unwinding in *M.tb* which contrasts that observed in *E. coli* where dATP and the non hydrolysable analog of ATP, ATPγS as well as CTP can substitute for ATP in open complex formation, but not UTP, GTP, dTTP and dCTP [1]. Unlike in *E. coli*, where ATP functions allosterically [10], in *M.tb* ATPase activity is also required. That ATP is critical for helix opening in *M.tb* is further supported by the observation that mutants defective in ATP hydrolysis were not viable [23]. Mutants which can bind ATP, but are unable to hydrolyze, are functionally similar to a situation of DnaA binding to ATPγS.

DnaA – ATP in *E. coli* is negatively regulated by Hda protein, by a process called RIDA (Regulatory Inactivation of DnaA). Hda and the β sliding clamp subunit (β clamp) of the DNA polymerase promotes hydrolysis of ATP bound to DnaA and thus inactivate DnaA [29]. Another mechanism of regulation of initiation involves the binding of many DnaA molecules to a chromosomal locus, *datA*, thereby reducing the number of DnaA molecules accessible to *oriC* [30], [31]. Both of these mechanisms perhaps do not operate in *M.tb*, as both *hda* gene and *datA* locus are absent. Therefore, the intrinsic ATPase activity of DnaA of *M.tb* may be critical in regulating replication in their absence.

**Figure 7:**
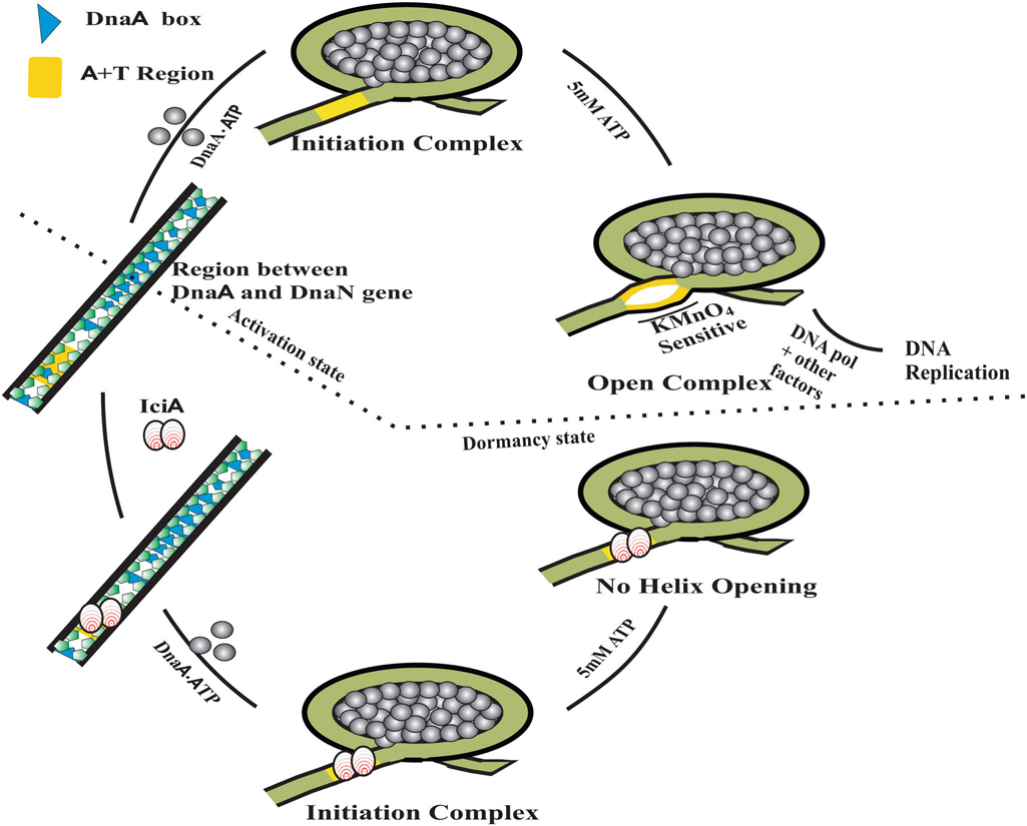
Schematic representation of the mechanism of helix opening by DnaA at *oriC* and its inhibition by IciA (modified from the model proposed by Bramhill and Kornberg [37] and Madiraju et al [23]). The ATP bound form of replication initiator DnaA protein binds to 13 DnaA boxes (darkly shaded arrow heads pointing the orientation of DnaA boxes) in *M.tb oriC* located between the *dnaA* and *dnaN* gene. Binding of DnaA-ATP complex to the DnaA boxes results in rapid oligomerization leading to the formation of the initiation complex (clockwise direction). Subsequently, the initiation complex gradually opens at A+T rich region which is then acted upon by a host of replication factors which finally lead to DNA replication. When IciA is present before the formation of open complex then it follows another pathway (anticlockwise direction). Here IciA protein binds to the A+T rich region of the *oriC*. In the presence of the DnaA protein the initiation complex is still formed however it eventually does not lead to the formation of open complex.

The putatively identified *M.tb* IciA, coded by ORF *Rv1985c*, inhibits helix opening as seen from KMnO_4_ probing experiments. By binding specifically to A+T region, as evident from EMSA (Figure 7), rIciA inhibits interaction between DnaA protein at the A+T rich region within the *oriC* – a process critical for helix opening in a manner similar to that seen in *E. coli* [14], [29]. Binding of rIciA consequently also inhibits *in-vitro* plasmid replication (Figure 5). DNA replication *in-vitro* using *M. bovis BCG* fraction II represents an authentic *in-vitro* enzyme system for studying replication involving *M.tb* origin. That rIciA is able to inhibit *in-vitro* DNA replication in this reconstituted system (Figure 5) clearly points to novel and an important role of IciA in inhibiting *M.tb* replication.

*E. coli iciA* null mutants are known to be completely viable and have the same growth rate as of wild type [12]. IciA is therefore not considered as a general replication inhibitor, but is thought to act under certain specific growth conditions. In *E. coli*, only limited sets of growth conditions have been evaluated and IciA and several other replication origin binding proteins may act as a replication inhibitor during nutrient starvation or during sudden changes in growth rate [15]. *M.tb* is known to survive for extended periods during the latency phase without any replication. During this phase bacteria sense the surrounding environmental conditions and *iciA* may have a role in maintaining mycobacterial latency. That IciA may have a role in *M.tb* latency is indirectly supported by results from *E. coli* where the concentration of IciA protein increases 4 fold (400 dimers per cell) as cells approach stationary phase [14] and cells which have elevated levels of IciA protein exhibit a growth lag upon transfer to fresh medium [12].

Based on our results we propose a working model for helix inhibition by IciA. The supercoiled template, having A+T rich region and 13 DnaA boxes, in the presence of DnaA protein and ATP binds to these DnaA boxes and causes rapid oligomerization of the supercoiled DNA. This interaction is favored by DNA bending proteins like HU. This is followed by the generation of open complex formation (Figure 7, upper half), so that other components of DNA replication can easily be loaded. Nearly about 19 nucleotides of the *oriC* region are unwound by DnaA alone, which can easily be detected by KMnO4 sensitivity of this region. The end product of this series of DNA protein interactions during *M.tb* chromosomal DNA replication signals the advent of the bacterial activation process. In contrast, during dormancy the IciA protein binds to the A+T rich region of the *oriC* (Figure 7, lower half) and this binding of IciA blocks DnaA dependent helix opening of the A+T rich region, a step critical for chromosomal initiation to occur. Consequently chromosomal DNA replication remains arrested so that *M.tb* can stay in a dormant state. It is therefore tempting to suggest that IciA could be one of the factor(s) involved in maintaining the latent state of growth of *M.tb.* Direct evidence for such a role of IciA will come from *M.tb iciA* knockouts in an infection model and also studies monitoring the steady expression level of *M.tb* IciA during latency and activation phase, in a clinical setting. While these experiments are underway, we are also investigating the quantitative expression of IciA as a molecular marker for *M.tb* activation.

## Materials and Methods

### Molecular cloning

The *M. tuberculosis* ORF *Rv1985c* and *Rv0001* coding for putative IciA protein and DnaA protein respectively, were PCR amplified using genomic DNA from H37Rv and primers IciAF, IciAR, DnaAF and DnaAR, carrying specific restriction enzyme sites (Table 1), by Accutaq DNA polymerase (Sigma). The amplicons thus generated were digested with *Nde*1/*Hin*dIII restriction enzymes and cloned into the corresponding sites of pET28a expression vector. The resultant plasmids were labeled as pETIciA and pETDnaA. For cloning intergenic region between *dnaA*/*dnaN* genes, the corresponding region was PCR amplified using MtbOriF and MtbOriR primer pair (Table 1). The amplicon thus generated was digested with *Hin*dIII/*Bam*H1 restriction enzyme and cloned into the corresponding site of pUC18 vector. The resultant plasmid was labeled as pUC_OriMtb. The authenticity of all constructs was confirmed by restriction analysis and DNA sequencing.

### Purification of recombinant His tagged IciA protein

Recombinant putative IciA, coded by *M.tb* ORF *Rv1985c*, was purified from the soluble fraction of BL21 (DE3) pLysS cells transformed with pETIciA grown overnight at 18°C and induced with 0.5 mM IPTG at an OD_600_ of 0.3 for the expression of recombinant protein as described earlier [32], [33]. The recombinant protein was purified in buffer containing 20 mM Tris, 300 mM NaCl and 10% glycerol. The purity of the protein was confirmed by SDS PAGE. The concentration of the protein was estimated by BCA (Bichinconic acid) and the purified protein was stored at −20°C until further use.

### Purification of recombinant His tagged DnaA protein

Recombinant DnaA protein was purified as described earlier [25] with minor modifications. To prevent the recombinant protein from getting complexed with ATP present in *E. coli* cytoplasm, which could interfere in the helix unwinding assays, the protein was denatured in buffer A [25 mM Tris acetate (pH 7.5), 250 mM NaCl, 0.1 mM EDTA, 10 mM Magnesium acetate and 10 mM β-mercaptoethanol] containing 8 M urea [23]. This was followed by sequential dialysis in 4 M, 2 M, 1 M and 0.5 M urea in buffer A containing 10% glycerol. The final dialysis buffer A contained 20% glycerol. The refolded DnaA protein, as seen on 10% SDS PAGE, was >95% pure. The protein concentration was estimated by BCA and stored at −20°C until further use.

### Preparation of fraction II

*In-vitro* replication competent fraction II was prepared by growing *M. bovis BCG Pasteur* in 600 ml of 7H9 media supplemented with OADC and casitone, in 1000 ml roller bottle at 37°C to log phase as described previously [34]. It took around 6–7 days for the cells to reach log phase from 1% primary inoculum. The cells were then harvested and resuspended in buffer B [25 mM, HEPES/KOH (pH 7.6), 0.1 mM EDTA, 2 mM DTT, and 100 mM potassium glutamate] supplemented with 1 mM PMSF. The cells were disrupted by sonication and the supernatant (fraction I) was precipitated by addition of ammonium sulphate (0.34 gm per ml of supernatant) with continuous stirring. This concentration was used for ammonium sulphate precipitation as it was known that *M.tb* DnaA precipitates with 34% ammonium sulphate cutoff. After an additional 30 min of stirring, the suspension was centrifuged at 4°C for 30 min at 18 000 g. The pellet was resuspended in minimal volume of buffer B (fraction II) of around 600 μl and dialyzed for 50 min at 4°C against 1000 fold excess of buffer B. Protein concentration was estimated by BCA and the replication competent fraction was flash frozen in small aliquots, so as to avoid freeze thaw, and stored at −70°C until further use. Each aliquot was used only once, after subsequent thawing the left over aliquot was discarded.

### ATPase activity

Reaction samples were kept on ice in 10 μl of buffer C [50 mM HEPES/KOH (pH 7.6), 0.5 mM Magnesium acetate, 2 mM DTT and 50 mM NaCl] containing 16 nM [γ^32^P]ATP and increasing amounts of DnaA protein as mentioned in figure legends. After incubating the samples for 30 min at 0°C, linear DNA carrying the DnaA box or pUC_OriMtb or pBSK II was added and the reactions were further continued at 37°C for 30 min. After this ATPase activity was determined by spotting 1.0 μl aliquot of each sample on Silica gel 60F_254_ thin layer chromatography plate (TLC). TLC plate was developed with chloroform: methanol: glacial acetic acid (65∶15∶5, v/v/v), followed by autoradiography and analyzing the image by Typhoon Variable Mode Imager and Image Quant software.

### Helix opening assay and KMnO_4_ probing

The standard helix opening assay (25 μl) was carried out in a buffer containing 40 mM HEPES-KOH (pH 7.5), 8 mM Magnesium acetate, 50 mM potassium glutamate, 1 μg poly dI/dC, 30% v/v glycerol, 320 μg/ml BSA and 550 ng supercoiled template (pUC_OriMtb), with indicated amounts of DnaA and or IciA (Rv1985) protein and 5.0 mM of either ATP or ADP or ATPγS (Lithium salt). The reaction mix was incubated for 30 min on ice followed by 20 min at 37°C. KMnO_4_ was then added to a final concentration of 10 mM, and the reaction was further continued for 2 min at 37°C. The reaction was stopped by the addition of stop buffer (1.75 M β mercaptoethanol and 50 mM EDTA) and samples were transferred to ice. 40 μl of phenol was then added and the samples were vortexed and centrifuged at 6000 rpm for 5 min. The supernatant was then passed through SephadexG50 spin column to purify the DNA template for use in primer extension reaction.

### Primer extension

10 μl of the primer extension mix included 200 μM each dNTPs, 0.04 pM ^32^P end labeled primer [SeqOriR1, SeqOriR2 or SeqOriR3 (Table 1)] 0.5 mM MgCl_2_, 2% DMSO and 0.5 Units Taq DNA polymerase (SIGMA). The mixture was subjected to primer extension (SeqOriR1) in a thermocycler for 30 cycles: 94°C for 1 min, 92°C for 30 sec., 54°C for 30 sec. and 72°C for 1 min except for 5 min in the last amplification cycle. All the conditions for primers SeqOriR2 and SeqOriR3 were identical, except that annealing was carried out at 48°C and amplification at 72°C for 40 sec. The reactions were stopped by adding 2 μl of formamide sequencing dye (95% Formamide, 10 mM NaOH, 0.05% Bromophenol blue and 0.05%Xylene Cyanol FF). The samples were heat denatured for 5 min at 95°C and subjected to 6% (or 15%) polyacrylamide gel electrophoresis containing 7 M urea. The gels were dried and analyzed by Typhoon Variable Mode Imager and Image Quant software.

### Assay for DNA replication

The standard reaction (20 μl), as described earlier [34], contained 40 mM HEPES.KOH (pH 7.6), 6 mM ATP, 500 μM of each GTP, CTP and UTP, 21.6 mM Creatine phosphate (Fluka), 50 μg/ml BSA, 100 μM each of dGTP, dCTP and dTTP, 50 μM dATP; 200 cpm/molar of total deoxynucleotide [α ^32^P]dATP, 11 mM Magnesium acetate, 35 μg Creatine Kinase (Sigma), 550 ng supercoiled plasmid DNA(pUC_OriMtb) and 7% PEG 10,000. All reactions were assembled on ice and started by the addition of 10–80 μg of protein (Fraction II or rIciA or both as indicated in figure legends) and incubating at 30°C for 30 min. Total nucleotide incorporation was measured by determining radioactivity retained after 10% trichloroacetic acid precipitation on nylon membrane through dot blot apparatus (BioRad). All the reactions were quantitated by Typhoon Variable Mode Imager and Image Quant software.

### Electrophoretic mobility shift assays

For electrophoretic mobility shift assays, synthetic complementary oligodeoxyribonucleotides OriF1 and OriR1 (Table 1) were annealed and 5′ end labeled using T_4_ Polynucleotide Kinase as described earlier [35], [36]. The ^32^P-labelled oligonucleotides were incubated with increasing concentration of IciA protein, at 30°C in binding buffer D [10 mM Tris (pH 7.5), 50 mM NaCl, 1 mM EDTA, 1 mM DTT, 50 μg/ml BSA, 1 μg poly dI/dC and 20% glycerol] for 30 min and the DNA-protein complex was fractionated on 5% native PAGE [0.25× TBE (22.25 mM Tris/borate/0.25 mM EDTA)] at 150 V, 4°C for 2–3 hrs. The gels were dried and analyzed by Typhoon Variable Mode Imager and Image Quant software.

## Supporting Information

Figure S1 IciA cannot inhibit helix unwinding once DnaA has already opened the DNA double helix. The reaction was carried out using 0.2 μg of DnaA protein. Helix opening was monitored by primer extension using SeqOriR1 on a 6% sequencing gel. The different lanes are: lane 3, With DnaA but without rIciA; lane 4: With DnaA and IciA (0.4 μg), but IciA was added after 10 min the addition of DnaA at 37°C. Arrows correspond to the extension products of 200 and 199 nucleotides. Lane 1 and 2 show 50 bp and 10 bp marker.
(1.05 MB EPS)
